# Lesão Miocárdica na COVID-19: Um Desafio para o Cardiologista Clínico

**DOI:** 10.36660/abc.20200434

**Published:** 2020-07-28

**Authors:** Roberto Cintra de Azevedo Aragão, Mariana Carvalho Alves, Hellen Dutra Passos, Luiz Flavio Galvão Gonçalves, Leonardo Baumworcel, José Augusto Soares Barreto

**Affiliations:** 1 Universidade Federal de Sergipe São CristovãoSE Brasil Universidade Federal de Sergipe, São Cristovão, SE - Brasil; 2 Hospital São Lucas Rede São Luiz D’or Centro de Ensino e Pesquisa AracajuSE Brasil Hospital São Lucas Rede São Luiz D’or - Centro de Ensino e Pesquisa, Aracaju, SE - Brasil

**Keywords:** Doenças Cardiovasculares, Dor torácica, Lesão Cardíaca, Miocardite, Coronavirus, COVID-19, Pandemia

Paciente masculino, 39 anos, sem comorbidades prévias, deu entrada no serviço de urgência com queixa de dor torácica de forte intensidade, associada a náuseas, sudorese e leve desconforto respiratório. A dor era de característica opressiva e irradiava para ambos os ombros. Relatava que há 2 dias iniciou quadro de astenia, inapetência e febre (38,9 ºC), evoluindo com lesões de pele eritematosas polimórficas no dorso, há 1 dia da admissão.

Exame físico revelou o seguinte: PA = 140/100 mmHg, FC = 90 bpm, afebril, SpO_2_ = 98% em ar ambiente, ritmo cardíaco regular em 2 tempos, sem sopros, pulmões limpos, com boa perfusão periférica e sem edema.

**Figura 1:**
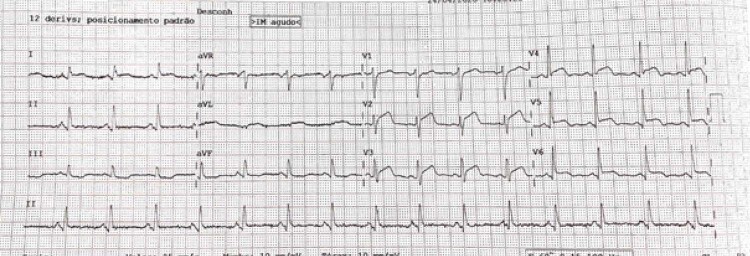
– *Eletrocardiograma com presença de supradesnivelamento do segmento ST, de V2 a V6.*

Eletrocardiograma evidenciou ritmo sinusal e supradesnivelamento do segmento ST nas derivações precordiais de V2 a V6 ( Figura 1 ).

Exames laboratoriais demonstraram o seguinte: troponina I = 25,20 ng/mL (valor normal [VN]: até 0,034 ng/ml), peptídeo natriurético cerebral = 1.460 pg/mL (VN: até 125 pg/ml), d-dímero = 104 ng/ml (VN: até 400 ng/ml), hemoglobina = 14,3 g/dl, leucócitos = 7.020 mm^3^ (78,1% neutrófilos e 9,7% linfócitos), plaquetas 145.000 e creatinina 0,6 mg/dl. As sorologias para HIV e citomegalovírus foram negativas, assim como a pesquisa do antígeno NS1.

O paciente recebeu dupla anti-agregação plaquetária com ácido acetilsalicílico 200 mg e ticagrelor 180 mg, sendo encaminhado para o serviço de hemodinâmica, onde foi submetido à cineangiocoronariografia que demonstrou coronárias com discretas irregularidades parietais difusas, isentas de ateromatose significativa.

Tomografia computadorizada de tórax evidenciou tênues áreas focais com opacidade em vidro fosco, isoladas na periferia do segmento basal posterior do lobo inferior direito (comprometimento < 25%), achado que pode ser visto em casos de pneumonia viral, porém não específico.

Ecocardiograma evidenciou hipocinesia do segmento médio da parede ântero-septal, com fração de ejeção preservada (62%) e mínimo derrame pericárdico difuso. As câmaras cardíacas estavam dentro dos limites da normalidade.

No terceiro dia após início dos sintomas, foi coletado swab oral e nasal para pesquisa de PCR-RT para COVID-19, que resultou positivo.

**Figura 2:**
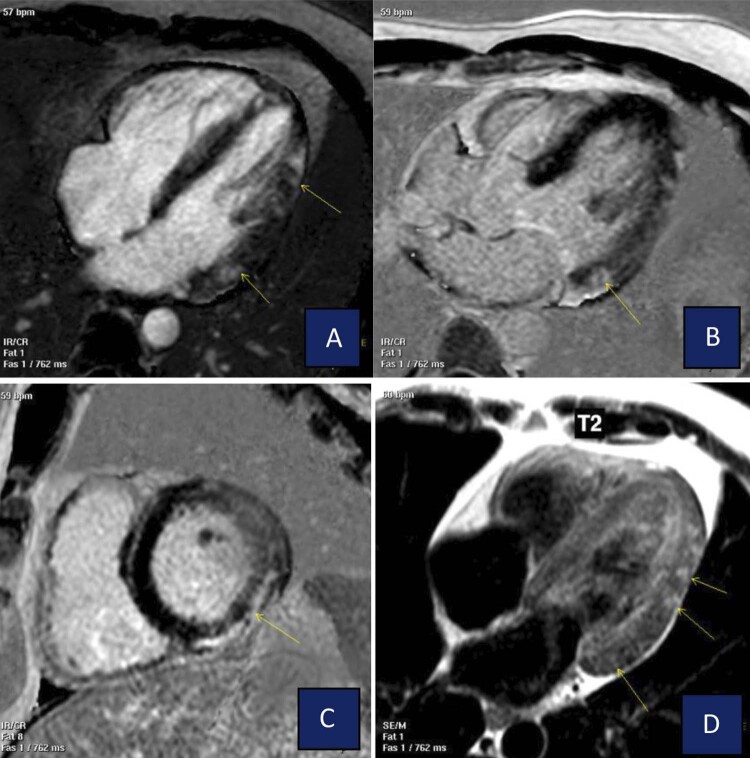
– *Sequências pós-contraste (A, B e C), demonstrando realce tardio meso-epicárdico, envolvendo as paredes inferior, ínfero-lateral e ântero-lateral, com discreta extensão para pericárdio adjacente e hipersinal associado nas sequências black-blood, com ponderação em T2 (D)*

Para elucidação diagnóstica do caso, optou-se por realizar ressonância magnética do coração que mostrou realce tardio meso-epicárdico envolvendo paredes inferior, ínfero-lateral e ântero-lateral, associado a hipersinal em T2, com discreta extensão para pericárdio adjacente, compatível com miopericardite aguda ( Figura 2 ).

O paciente foi internado inicialmente na unidade de terapia intensiva. Evoluiu em bom estado geral, assintomático e recebeu alta hospitalar no oitavo dia, em uso de betabloqueador e inibidor do receptor da angiotensina AT1.

Diante da pandemia pelo novo coronavírus, já é possível evidenciar a correlação entre a COVID-19 e as complicações cardiovasculares desta doença.^1 , 2^ Nesse contexto, o acometimento cardiovascular como condição de alta morbi-mortalidade vem se mostrando com grande variabilidade de apresentações, sobrepondo-se às manifestações da COVID-19,^3 - 5^ sendo então necessária a avaliação cardiológica inicial e regular no seguimento dos pacientes infectados, a fim de minimizar desfechos desfavoráveis.

No caso em questão, foi observado que um paciente jovem, sem fatores de risco, também pode ser alvo de complicações cardíacas no curso da infecção pelo novo coronavírus. Ainda serão necessários maiores estudos para que haja melhor elucidação dos fatores preditores e desfechos deste acometimento.
