# Supplementary material for: Lesão Miocárdica na COVID-19: Um Desafio para o Cardiologista Clínico
Source: Arq Bras Cardiol. 2020 Jul 28;115(1):139–41. [Article in Portuguese] doi: 10.36660/abc.20200434 (PMC8384318; doi:10.36660/abc.20200434)
Supplement: Translation

# Translation: Myocardial Injury in COVID-19: a Challenge for Clinical Cardiologists

**Authors:** Roberto Cintra de Azevedo Aragão, Mariana Carvalho Alves, Hellen Dutra Passos, Luiz Flavio Galvão Gonçalves, Leonardo Baumworcel, José Augusto Soares Barreto

**Affiliations:** 1 Brazil Universidade Federal de Sergipe, São Cristovão, SE - Brazil; 2 Brazil Hospital São Lucas Rede São Luiz D’or - Centro de Ensino e Pesquisa, Aracaju, SE - Brazil

**Keywords:** Cardiovascular Diseases, Chest Pain, Cardiac Injury, Myocarditis, Coronavirus, COVID-19, Pandemic

A 39-year-old male patient, with no previous comorbidities, was admitted to the emergency department presenting intense chest pain, associated with nausea, sweating, and mild respiratory distress. The pain was oppressive, and it radiated to both shoulders. The patient reported onset of asthenia, lack of appetite, and fever (38.9 ºC) 2 days prior, and he presented with polymorphic erythematous skin lesions on his back 1 day after admission.

Physical examination revealed the following: blood pressure = 140/100 mmHg, heart rate = 90 bpm, no fever, SpO_2_= 98% in room air, regular cardiac rhythm with 2 beats, no murmurs, clean lungs, good peripheral perfusion, and no edema.

Electrocardiogram revealed sinus rhythm and ST-segment elevation in precordial leads V2 to V6 ( Figure 1 ).

**Figure 1:** – *Electrocardiogram showing the presence of ST-segment elevation on leads V2 to V6.*

Laboratory exams revealed the following: troponin I: 25.20 ng/mL (normal value [NV]: ≤ 0.034 ng/ml), brain natriuretic peptide (NT-ProBNP): 1,460 pg/mL (NV: ≤ 125 pg/ml), D-dimer 104 ng/ml (NV: ≤ 400 ng/ml), hemoglobin 14.3 g/dl, leukocytes 7,020 mm^3^(78.1% neutrophils and 9.7% lymphocytes), platelets 145,000, and creatinine 0.6 mg/dl. Sorology for HIV and cytomegalovirus were negative, as was NS1 antigen test.

The patient was started on dual antiplatelet therapy with acetylsalicylic acid 200 mg and ticagrelor 180 mg, and he was referred to the hemodynamic service, where he underwent coronary cineangiography, which showed coronary arteries with mild diffuse parietal irregularities, without significant atheromatosis.

Chest computed tomography showed tenuous focal areas with ground-glass opacity, isolated on the periphery of the posterior basal segment of the right lower lobe (impairment < 25%). This finding may be observed in cases of viral pneumonia; it is, however, non-specific.

Echocardiogram showed hypokinesia of the middle segment of the antero-septal wall, with preserved ejection fraction (62%) and minimal diffuse pericardial effusion. Cardiac chambers were within normal limits.

On the third day after onset of symptoms, oral and nasal swabs were collected for RT-PCR for COVID-19, the results of which were positive.

To clarify the diagnosis, cardiac magnetic resonance imaging was performed, which showed meso-epicardial late enhancement in the inferior, inferolateral and anterolateral walls, associated with hypersignal in T2, with slight extension to the adjacent pericardium, compatible with acute myopericarditis ( Figure 2 ).

**Figure 2:** – *Post-contrast sequences (A, B, and C), demonstrating meso-epicardial late enhancement, involving inferior, inferolateral and antero-lateral walls, with slight extension to the adjacent pericardium and associated hypersignal, in black-blood sequences, predominantly in T2 (D).*

The patient was initially admitted to the intensive care unit. He progressed in good general condition; he was asymptomatic, and, on the eighth day, he was discharged, using a betablocker and an AT1 angiotensin receptor blocker.

Faced with the novel coronavirus pandemic, it has already been possible to see evidence of the correlation between COVID-19 and cardiovascular complications caused by this disease.^1 , 2^ In this context, cardiovascular involvement, a condition with high morbimortality, has shown great variability regarding clinical presentation, overlapping with manifestations of COVID-19,^3 - 5^ thus making initial cardiological evaluation and regular follow-up of infected patients necessary in order to minimize unfavorable outcomes.

This case indicate that young patients without risk factors may also suffer from cardiac complications during the course of infection with the novel coronavirus. Larger studies will be necessary in order to further clarify predictive factors and outcomes related to cardiac involvement.
